# Dynamic Enhancement of Nitric Oxide Radioluminescence with Nitrogen Purge

**DOI:** 10.1038/s41598-019-50396-6

**Published:** 2019-09-25

**Authors:** Thomas Kerst, Juha Toivonen

**Affiliations:** 1 Photonics Laboratory, Physics Unit, Tampere University, Tampere, Finland; 2 Helsinki Institute of Physics, Helsinki University, Helsinki, Finland

**Keywords:** Optics and photonics, Applied optics, Optical techniques

## Abstract

Remote detection of alpha radiation is commonly realised by collecting the light, the radioluminescence, that is produced when alpha particles are stopped in air. Radioluminescence of nitric oxide (NO) is primarily emitted between 200 nm and 300 nm, which makes it possible to use it for remote detection under daylight conditions. Quenching by ambient oxygen and water vapour, however, makes it generally difficult to effectively create NO radioluminescence. We present the detection of intense NO radioluminescence in ambient air under standard indoor lighting conditions using a nitrogen purge. The nitrogen contained NO impurities that were intrinsic to the gas and had not explicitly been added. We study the mechanisms that govern the NO radioluminescence production and introduce a model to describe the dynamics of the process. The level of NO contained in the gas was found to determine how successful a purge can be. We conclude by discussing possible applications of the technique in nitrogen-flushed gloveboxes at nuclear facilities where NO concentration of 100 ppb–1 ppm would be sufficient for efficient optical alpha radiation detection in standard lighting conditions.

## Introduction

An alpha particle that is being stopped in air causes the production of light. Upon decay, an atomic nucleus loses energy by emitting a combination of alpha particles, beta particles, and gamma radiation. All three forms of radiation are ionising, which means that they can detach electrons from atoms or molecules. Electrons freed through ionisation deposit their energy into the matter they interact with, which can cause it to become electronically excited^1,2^. When this excitation is lost in a radiative process, then the generated light is called radioluminescence rather than fluorescence. The radioactive decay had to occur for the photon to be generated, and the name highlights this fact.

Radioluminescence is a useful indicator for the presence of alpha radiation. Unlike other forms of nuclear radiation, alpha radiation is very localised. Alpha particles do not travel further than about $4{\rm{cm}}$ in air, whereas beta and gamma radiation can reach distances of about 1 m and 50 m, respectively^3^. All ionising radiation can create radioluminescence. However, alpha radioluminescence is special in that it travels much farther than the alpha particles themselves. This gives any alpha radiation detection scheme based on radioluminescence two key advantages over those based on direct interaction. The scheme works at much larger distances, and equipment does not need to be exposed to the particles themselves, which present a particularly large hazard to both personnel and equipment^4,5^. In short, it enables the remote detection of alpha radiation from a safe distance. In the rest of this article, we focus exclusively on alpha radiation-induced radioluminescence and address it by the shorthand “radioluminescence”.

In ambient air, nitrogen (N_2_) radioluminescence is a reliable indicator for the presence of alpha radiation. Alpha particles create free electrons which then electronically excite nitrogen molecules^1^. Those excitations that radiatively decay over the ${{\rm{C}}}^{3}{\Pi }_{{\rm{u}}}\to {{\rm{B}}}^{3}{\Pi }_{{\rm{g}}}$ transition cause the emission of photons whose spectral pattern is commonly referred to as ‘Second Positive System’ (or ‘2+’ in short), which primarily consists of ultraviolet light emitted between 320 and 400 nm and a notably strong peak around 337 nm^3,6,7^. A small fraction of the N_2_ radioluminescence is emitted in the deep ultraviolet regime, where it reaches wavelengths as short as 260 nm^8^. In principle, the 2+ comprises light with even shorter wavelengths, however the shortest wavelengths used so far to remotely detect alpha radiation have been around 260 nm^6,8^. The spectral pattern of N_2_ radioluminescence is very characteristic, and it is generally accepted that under normal circumstances detecting light emitted on the 2+ system is a good indicator for the presence of alpha radiation.

Radioluminescence in air is very weak compared to other light sources, such as the sun. A single alpha particle stopped in air produces of a small amount of roughly 100 UV photons which are isotropically radiated away^3,7^. Consequently, even strong alpha sources provide few photons that can be used for remote detection at reasonable distances. For this reason, radioluminescence detection schemes generally rely on technology capable of detecting individual photons^5^. For instance, detecting N_2_ radioluminescence with a photomultiplier tube (PMT) in a UV-background lighting free environment is generally possible. In a recent contribution, a PMT equipped with optimised light collection optics was able to resolve sources with surface activities as low as 300 Bq mm^−2^ at 1 m distance^8^. Some cameras, too, are sufficiently sensitive to detect radioluminescence. In recent years a sizable body of works has accumulated reporting on the successful imaging of alpha radiation using various types of specialised cameras like ICCD and EMCCD^4,9–11^. Both types of collecting radioluminescence have made it possible to detect alpha radiation in difficult-to-access areas like gloveboxes and hot-cells from a safe distance^10,12,13^.

The presence of UV background light makes N_2_ based remote detection difficult. By in large, the remote detection schemes demonstrated so far rely on the assumption that the presence of UV light is an indicator good enough to potentially reveal the presence of alpha radiation. If, however, any other UV light source other than radioluminescence is present, this assumption no longer holds. Some of the presented technologies tackle this problem by tailoring the detection optics to only transmit light of wavelengths around 337 nm while rejecting all others^8^. However, a UV background at just those wavelengths can supersede the faint radioluminescence. Sunlight reaching the surface of the earth is such a light source^14^. It is for this reason that remote detection of alpha radiation with sunlight present is a much more challenging task than without it^8,15^.

One way to make N_2_ radioluminescence detection operable in daylight is by making the detection optics solar blind. Sunlight that is emitted between 100 to 280 nm is absorbed by ozone in the upper atmosphere and does not reach the earth’s surface^14,16^. This part of the electromagnetic spectrum is called the UVC region and its essential characteristic is the absence of sunlight that reaches the earth surface. Making the optics unresponsive to photons of wavelengths longer than 280 nm, that is to make it solar blind, while leaving it responsive to the UVC adequately addresses the issue. Sand *et al*. successfully built and demonstrated such a system in a field environment using a caesium-telluride PMT and a set of bandpass filters centred around 260 nm^8^. Another successful demonstration of an optical system with a tailored response was by Ivanov *et al*. who imaged N_2_ radioluminescence using a UVC-sensitive camera that at the same time was insensitive to sunlight^17^. However, all those approaches suffer from the fact that only a small portion of N_2_ radioluminescence is located in the UVC region^18^. Restricting the optical response of the detection system to the UVC limits its ability to detect weaker alpha sources. By using solar blind optics, the detection system gains the ability to operate under daylight conditions but at the same time severely reduces its sensitivity.

Another way to address a daylight background is to use radioluminescence from molecules other than N_2_. A wide variety of species have been shown to produce radioluminescence, where OH, CN, NH, CO_2_, ${{\rm{N}}}_{2}^{+}$, He_2_, O^−^ are only a few of them^19–21^. Most of them, however, are generally irrelevant for detecting alpha particle radiation since they are commonly not present where remote detection would be required. In most cases, alpha contamination is surrounded by air, which typically limits the useful species to N_2_. It is the only species that both is sufficiently abundant and at the same time produces radioluminescence that it is not as strongly affected by O_2_ and H_2_O quenching like radioluminescence from most other species is^18,22–25^. If, however, one can alter the atmosphere around an alpha emitter, then one is no longer limited to relying on N_2_ as luminescent species. In a recent study, Crompton and colleagues tested a few known radioluminescent species for their aptitude to be used in remote detection of alpha radiation^15^. Among others, they found that xenon is a strong candidate that could potentially be used to address the daylight issue.

Another molecule that is potentially useful as luminescent species is nitric oxide (NO). NO can produce radioluminescence that is almost exclusively located in the UVC by forming the so-called NO *γ*-band between 200 nm and 300 nm^26^. Its advantageous spectrum combined with its ability to quickly re-excite after a radiative decay by interaction with N_2_ makes NO a strong candidate^27^. A nitrogen molecule that has been excited by the presence of an alpha source transfer its excitation to a nitric oxide molecule, where it radiatively decays as radioluminescence. This process is very efficient and thus introducing some nitric oxide to an otherwise pure nitrogen atmosphere leads to the production of large amounts of UVC radioluminescence. Adding as little as 50 ppm of NO alpha emitter can increase the overall radioluminescence yield 25-fold, where almost all of the so-created radioluminescence is located in the UVC^28^. The conditions under which this can be achieved have to be very controlled and potent quenchers like O_2_ and H_2_O have to be absent^22,23,28^. In ambient air, where NO is present in concentrations ranging between tens of ppt and tens of ppb^29^, those quenchers are very abundant which renders nitric oxide radioluminescence generally unpractical. However, if conditions can be created that favour its production, nitric oxide radioluminescence is a viable option to generate intense radioluminescence in the UVC.

In this paper, we present radioluminescence emanating from nitric oxide impurities in otherwise pure nitrogen and study the conditions under which it arises. We demonstrate that higher levels of impurities not only increase the overall intensity of the radioluminescence but also speed up the process that maximises its production rate. We show that the predictions made by the model of NO radioluminescence production based on excitation transfer match with the predictions made by more established theories. We provide further evidence for the plausibility of the model by showing that a simulation which is based on a description with excitation transfer can replicate experimental findings. We experimentally demonstrate that a nitrogen purge with adequate levels of impurities can produce large amounts of UVC photons around an alpha emitter placed in ambient air. We conclude our work by discussing the usefulness of a nitrogen purge in nuclear research facilities.

## Methods

**Figure 1:**
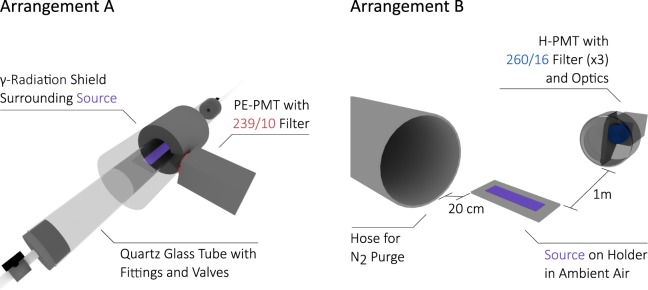
The experimental arrangements used in this work. In **Arrangement A** we placed a 32 MBq alpha source on glass holder resting inside a quartz glass tube. A UVC-sensitive PMT equipped with a 239/10 optical bandpass filter picked up radioluminescence emanating inside it. A metal shield around the tube protected both personnel and equipment from *γ* radiation. The glass tube was connected to a system of mass flow controllers that allowed to flush with either a N_2_(NO: 5 ppb) or N_2_(NO: 50 ppb) at different flow rates. Valves allowed to prevent gas exchange with the laboratory air. In **Arrangement B**, we placed the same alpha source in ambient air and installed a large diameter hose in 20 cm distance to it. The hose provided a purge with either N_2_(NO: 5 ppb) or N_2_(NO: 50 ppb). A UVC-sensitive PMT equipped with a stack of three 260/16 optical bandpass filters and an adequate objective was placed at a distance of about 1 m and picked up light emanating from the source.

All experiments were carried out in one of two distinct arrangements which are sketched in Fig. 1. With Arrangement A, we studied the dynamics of NO radioluminescence production by placing an alpha emitter in a glass tube where we precisely controlled the gas atmosphere. With arrangement B, we studied how efficiently UVC radioluminescence can be generated in ambient air by placing the same emitter in ambient air and applying a nitrogen purge. In both arrangements, we used the same 32 MBq alpha active Am-241 source. The active area of the source was 50 mm in length and 12.5 mm in width. In both arrangements, we used nitrogen gas to modify the radioluminescence production (AGA, Product Codes: Instrument Nitrogen 5.0 and Scientific Nitrogen 6.0). Instrument Nitrogen 5.0 contained impurities up to a total volume concentration of 10 ppm and a NO volume concentration of 5 ppb, to which we refer by using the shorthand N_2_(NO: 5 ppb). Scientific Nitrogen 6.0 contained impurities up to a total volume concentration of 1 ppm and a NO volume concentration of 50 ppb, to which we refer by using the shorthand N_2_(NO: 50 ppb). The NO concentrations had been verified using a chemiluminescence analyser^30^ which we calibrated using a N_2_ gas sample containing a known NO concentration.

### Radioluminescence dynamics

**Figure 2:**
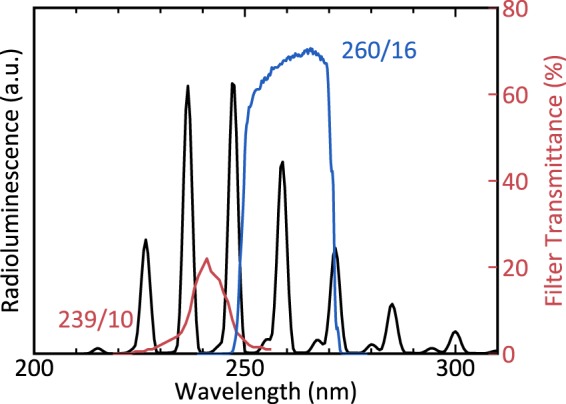
The spectrum of NO radioluminescence and the transmittance of the bandpass filters used in this work. NO produces radioluminescence (black, taken from Kerst *et al*.^28^) that appears in distinct spectral lines between 200 nm and 320 nm^26^. The optical filters used for either setup only transmitted parts of the spectrum. Arrangement A used a filter (red) that transmitted light around 239 nm with a FWHM of 10 nm and a peak transmittance of about 20%^31^. Arrangement B used a stack of three filters (blue), each of which transmitted light around 260 nm with a FWHM of 16 nm and a peak transmittance of about 70%^33^. The stack of all three of these filters had a peak transmittance of about 30%.

With arrangement A, we studied the dynamics of radioluminescence production by placing the source in a quartz glass tube and controlled the atmosphere inside with a set of mass flow controllers and valves. The quartz glass tube was 1 m long, had an outer diameter of 30 mm and 1.5 mm thick walls (Robson Scientific, Product Code: RQT 30). The source rested on a rectangular sample holder made of glass at the centre of the tube. A 21 mm thick steel mantle surrounded the glass tube, protecting personnel and equipment from gamma radiation. A cone-shaped hole with the f-number *f*/1.378 in the steel mantle allowed light originating close to the source to escape the tube and be collected by a UV-sensitive photomultiplier tube (PMT, Perkin-Elmer, Product Code: MP-1082, Dark Count: <1 cps). An optical bandpass filter between PMT and tube only transmitted light around the wavelength 239 nm with a FWHM of 10 nm (Edmund Optics, Product Code: 67805, transmittance shown in Fig. 2 in red)^31^. By using a filter with a transmittance spectrum deep in the UVC, we made sure that we only studied the dynamics of NO radioluminescence. Some of the NO radioluminescence could pass the filter and reach the detector, while radioluminescence of N_2_ was prevented from doing so. We controlled the atmosphere inside the tube with two mass flow controllers (MFC, Bronkhorst High-Tech, Product Codes: 18BRF-201CV-10K and 18BRF-201CV-20K), where each controller had access to either type of used nitrogen. We sealed the tube with custom fittings to shield the atmosphere inside the tube from laboratory air and made sure that the tube was only connected to the MFCs, and a gas exhaust and mechanical valves (Swagelok, Product Code: SS-6P4T-MM-BK). All tubings were kept as short as possible to prevent the formation of large dead volumes.

We studied the dynamics of radioluminescence production by monitoring the changes in radioluminescence intensity when a flow of nitrogen that had been applied for 30 min was suddenly stopped. In each experiment we flushed the laboratory air-filled tube with either 1 SLPM, 2 SLPM or 4 SLPM of either N_2_(NO: 5 ppb) or N_2_(NO: 50 ppb). Then we stopped the flow, closed the valves and continued to monitor the radioluminescence intensity for another 10 min. Before experimenting, we removed the glass tube from the setup and exposed it to normal laboratory air for at least 2 hours. This way we made sure that all flow experiments were carried out with identical initial conditions: The initial atmosphere inside the tube was always ambient laboratory air, and the initial amount of water present at the inside tube walls was always the same. We took those measures to address the problem of changing water concentrations during our experiments. Water forms a thin film on glass surfaces^32^, and it was challenging to remove this film to the degree that would be required to make radioluminescence quenching negligible^23^. At the same time, it was difficult to keep the water concentration constant during the experiment, such that water quenching would reduce the overall NO radioluminescence but not change the observed dynamics that we intended to study. Flushing slowly but gradually removed water from the walls, making it impossible to keep the water concentration constant. Thus it was sensible to address the issue by considering the influence of water when post-processing the data and making sure that each experiment was carried out with identical initial conditions.

### Remote detection

With arrangement B we studied how useful of a nitrogen flush can be in producing NO radioluminescence in otherwise ambient air. For this experiment, we placed the source in ambient air under normal lighting conditions. We placed a hose with 10 cm diameter in 20 cm distance to it which was able to provide a purge using either type of nitrogen. In 1 m distance we placed a caesium-telluride photocathode PMT (Hamamatsu, Product Code: H11870-09) and equipped it with an objective made of standard UV fused silica lenses as described in Sand *et al*.^8^ a stack of three identical bandpass filters that transmitted light around 260 nm with an FWHM of 16 nm (Semrock, Product Code: FF01-260/16-25, transmittance of a single filter shown in Fig. 2 in blue)^33^. The filters chosen for this arrangement had peak transmittances superior to this in arrangement A and were better suited to detect increases in radioluminescence production in ambient air. We carried out an experiment by applying a purge of about 100 SLPM of either N_2_(NO: 5 ppb) or N_2_(NO: 50 ppb) for about 5 seconds. At all times, we measured the UVC light emanating from the source.

## Results and Discussion

### Radioluminescence dynamics

**Figure 3:**
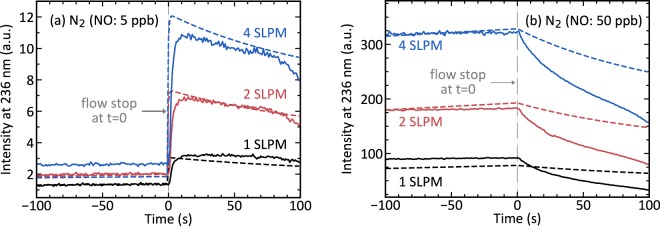
The dynamics of NO radioluminescence in the tube once a previously applied nitrogen flow had been stopped. A 30 min lasting nitrogen mass flow of 1, 2 or 4 SLPM of either (**a**) N_2_(NO: 5 ppb) or (**b**) N_2_(NO: 50 ppb) had been stopped and the valves closed at *t* = 0. For N_2_(NO: 5 ppb) gas, stopping the flow caused an immediate jump-like increase in the radioluminescence, whereas for N_2_(NO: 50 ppb) gas it did not. For both gases, radioluminescence gradually decreased afterwards. Solid lines show the measured data and dashed lines the best fit of the simulation to it.

Figure 3 shows the dynamics of the radioluminescence we observed upon stopping the nitrogen flow. Flushing with N_2_(NO: 50 ppb) produced significantly more radioluminescence than flushing with N_2_(NO: 5 ppb) and flushing with more litres per minute produced more radioluminescence than flushing with fewer. After stopping the flow, the radioluminescence intensity jump-like increased for N_2_(NO: 5 ppb) but not for N_2_(NO: 50 ppb). For both gases, however, the intensity gradually decreased after the flow had been stopped. We hypothesise this decrease to be caused by quenching by water vapour, which diffused into the optical volume. In the absence of a nitrogen flow, water vapour no longer got flushed out and could concentrate in the optical volume. A concentration of water vapour could also explain the observation that a larger mass flow resulted in more radioluminescence production since a larger mass flow provided more nitrogen in which water vapour could be diluted into, thus reducing the effects of quenching. Water quenches nitric oxide radioluminescence very efficiently, and even ppm-levels of water vapour have a significant effect on the radioluminescence intensity^22,23^.

The more intense radioluminescence produced by a N_2_(NO: 50 ppb) flush can not be explained with a higher amount of nitric oxide alone. N_2_(NO: 50 ppb) contained 10 times more nitric oxide than N_2_(NO: 5 ppb). Thus, it is sensible to assume that under equilibrium conditions N_2_(NO: 50 ppb) produces significantly more radioluminescence, given that nitric oxide with concentrations that small exhibits negligible self-quenching^23^. However, the data reveals that the increase in radioluminescence production is different whether the flow had been stopped or not. Before stopping, the radioluminescence was about 100 times and after stopping about 30 times more intense, irrespective of whether the intensities for a mass flow of 1, 2 or 4 SLPM are compared. We, therefore, conclude that before the flow had been stopped, radioluminescence produced by N_2_(NO: 5 ppb) had not fully developed, and it can not be assumed that the system was in equilibrium.

More careful consideration of the processes that lead to the production of nitric oxide radioluminescence offers a better explanation. NO radioluminescence is produced as the last step in a chain of three consecutive events: first alpha radiation excites surrounding molecular nitrogen into the long-lived ${{\rm{A}}}^{3}{\Sigma }_{{\rm{u}}}^{+}$ state, then this excitation is transferred to ground state nitric oxide where it causes the ${{\rm{A}}}^{2}{\Sigma }^{+}$ state to be populated and lastly this excitation radiatively decays by emitting a photon^26,28^. Nitric oxide decays with a natural lifetime of approximately 200 ns^34^, which is considerably shortened in the presence of potent quenchers like water vapour and molecular oxygen^22,23^. Thus, the mechanism that limits the speed by which NO radioluminescence is produced is the speed by which the excitations are transferred. The rate equation^28^

1 $$\frac{{\rm{d}}}{{\rm{d}}t}[{{\rm{N}}}_{2}^{{\rm{A}}}]=\alpha [{{\rm{N}}}_{2}^{{\rm{X}}}]-{k}_{{\rm{f}}}[{{\rm{N}}}_{2}^{{\rm{A}}}]-{k}_{{\rm{et}}}[{{\rm{NO}}}^{{\rm{X}}}][{{\rm{N}}}_{2}^{{\rm{A}}}]$$

describes this transfer, where $[{{\rm{N}}}_{2}^{{\rm{X}}}]$, $[{{\rm{N}}}_{2}^{{\rm{A}}}]$ and [NO^X^] are the concentrations of ground state nitrogen, excited state nitrogen and ground state nitric oxide, respectively. *α* is the rate by which the alpha radiation excites nitrogen and ${k}_{{\rm{et}}}[{{\rm{NO}}}^{{\rm{X}}}]$ the rate by which those excitations are transferred to ground state nitric oxide. Nitrogen fluorescence is with a rate of *k*_f_ ≈ 0.4 Hz^6^ very slow compared to excitation transfer and thus negligible. Further, the short lifetime of excited nitric oxide makes it that most of the nitric oxide is present in the ground state, i.e. one can approximate $[{{\rm{NO}}}^{{\rm{X}}}]\approx [{\rm{NO}}]$, where [NO] the total nitric oxide concentration. Such an approximation allows to express the excited state nitrogen concentration $[{{\rm{N}}}_{2}^{{\rm{A}}}]$ after a time *t* after first exposure to the alpha source with a simple exponential function

2 $$[{{\rm{N}}}_{2}^{{\rm{A}}}](t)={[{{\rm{N}}}_{2}^{{\rm{A}}}]}_{\infty }(1-{e}^{-\gamma t}),$$

where ${[{{\rm{N}}}_{2}^{{\rm{A}}}]}_{\infty }$ is the equilibrium concentration and $\gamma ={k}_{{\rm{et}}}[{\rm{NO}}]$ the speed by which this equilibrium is approached. In this model the rate of excitation transfer limits the rate by which radioluminescence is produced, thus the radioluminescence intensity *I*(*t*) is proportional to the excited state nitrogen concentration $[{{\rm{N}}}_{2}^{{\rm{A}}}](t)$. With this model, the jump-like behaviour of N_2_(NO: 5 ppb) induced radioluminescence can be understood as a result of the excited state nitrogen not having reached equilibrium concentration during the short amount of time it was exposed to the source during flushing. For a NO concentration of 5 ppb the model estimates a rise-time of roughly 90 ms for the radioluminescence to reach half its maximum intensity and 600 ms to reach 99%. With flows in the range of litres per minute, the time the gas is exposed to the source is a few hundreds of milliseconds or less, which does not allow the gas to reach maximum radioluminescence production. After stopping the flow, however, it had sufficient time and eventually reached maximum intensity. According to the model, this happens in less than a second, which is in agreement with the measured data. N_2_(NO: 50 ppb) contained much more nitric oxide and, according to the model, approached the equilibrium much faster. Thus stopping the flow and exposing it longer to the source did not have as much of an effect as it had for N_2_(NO: 5 ppb).

Our interpretation of the data based on excitation transfer makes predictions about the experiment that match those made by more established theories. In Eq. (2), our model links the radioluminescence intensity of a partially evolved system to this of a fully evolved one using the excitation transfer rate and the time *t*_0_ the gas is exposed to the source. By applying this to the stop-flow behaviour of the radioluminescence of N_2_(NO: 5 ppb), the exposure time as a function of the mass flow can be estimated. The relation between mass flow and exposure time can be tested for its compliance with the fundamental principles of fluid dynamics. To properly apply the model to the measured data, it has to be adjusted to include the fact that the PMT picked up light emanating from all parts of the source. Radioluminescence produced at the start of the source is less intense than this produced at the end of it. By approximating all parts to equally contribute to the total intensity, irrespective of their actual distance to the PMT, the intensity measured by the PMT can be expressed by calculating the simple average and a multiplying with a geometry-specific scaling constant *C*, which is to say

3 $${I}_{{\rm{PMT}}}({t}_{0})=C\cdot \frac{1}{{t}_{0}}{\int }_{0}^{{t}_{0}}I(t){\rm{d}}t=C\cdot {I}_{\infty }(1-\frac{1-{e}^{-\gamma {t}_{0}}}{\gamma {t}_{0}}).$$

**Figure 4:**
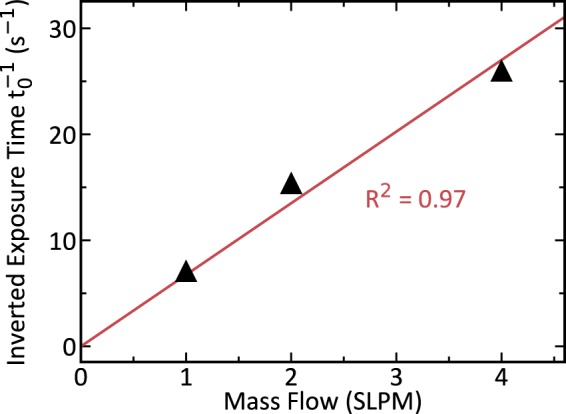
The estimated time the N_2_(NO: 5 ppb) is exposed to the source displayed inverted against the mass flow. The estimates (black) have been calculated by numerically solving Eq. (3) using data from Fig. 3(a). A linear fit crossing the coordinate origin (red) with goodness of fit R^2^ = 0.97 shows that the exposure time is inversely proportional to the mass flow.

We numerically solved this equation for the exposure time *t*_0_ for all three mass flows *Q*. For *I*_PMT_(*t*_0_) we used radioluminescence values from Fig. 3(a) at *t* < 0 and for *C* · *I*_*∞*_ values at *t* > 0. We picked values around *t* = 0 to ensure that the amount of water in the tube at both points in time was similar. The so-calculated inverted exposure times are plotted against the mass flow in Fig. 4. There it can be noticed that exposure time and mass flow are inversely proportional to each other, which we highlighted by adding a linear regression with zero offset and quality of fit *R*^2^ = 0.97. This inverse relationship is congruent with the predictions of fluid dynamics. Nitrogen gas is a Newtonian fluid and as such travels over the length of the source in a time *t*_0_ ∼ *Q*^−1^, if the flow is laminar^35^. In our system, the Reynolds numbers for all flows were well below the values for which transitions to turbulent flows might start to occur^36^.

We further tested how well our model can replicate the dynamics of the stop-flow behaviour by fitting a simulation of the experiment to the measured data. The simulation is based on the model of NO radioluminescence production described by Kerst *et al*.^28^. This description consists of a set of rate equations which we amended by a second set of rate equations that modelled the changing water concentration in the tube. We solved the first set of equations using an ODE solver^37^ where we simplified the calculation by assuming that all layers of the gas over the source moved at identical speed and were equally exposed to alpha radiation, much as it has been done in Eq. (3). All rate equations had been amended to account for quenching by water using known quenching constants^22,23,38^. The geometry of the system, filter transmittance and PMT quantum efficiency have been included using a global scaling constant. We modelled the water dynamics as simple as possible but still able to include diffusion and effects of flushing. For that, we approximated the water to be present in one of two distinct reservoirs. Reservoir I represented the optical volume, and water present here quenched the radioluminescence. When a mass flow was present, the water concentration lowered and quenching reduced. Reservoir II represented the parts of the system that were unaffected by flushing, like glass walls and other adhesive surfaces. We modelled it to exchange water with reservoir I using ordinary linear differential equations of the form

4 $$\frac{{\rm{d}}}{{\rm{d}}t}{[{{\rm{H}}}_{2}{\rm{O}}]}_{{\rm{wall}}}=-{k}_{{\rm{diff}}.}{[{{\rm{H}}}_{2}{\rm{O}}]}_{{\rm{wall}}}+{k}_{{\rm{adh}}.}{[{{\rm{H}}}_{2}{\rm{O}}]}_{{\rm{vol}}.}$$

5 $$\frac{{\rm{d}}}{{\rm{d}}t}{[{{\rm{H}}}_{2}{\rm{O}}]}_{{\rm{vol}}.}={k}_{{\rm{diff}}.}{[{{\rm{H}}}_{2}{\rm{O}}]}_{{\rm{wall}}}-{k}_{{\rm{adh}}.}{[{{\rm{H}}}_{2}{\rm{O}}]}_{{\rm{vol}}.}-{k}_{{\rm{flow}}}\Phi {[{{\rm{H}}}_{2}{\rm{O}}]}_{{\rm{vol}}.},$$

where Φ is the mass flow in SLPM. All parameters introduced with this simple model were free for the optimiser to tune.

We optimised the parameters such that the simulation best fits all six measured curves all at once^39^, which means that all simulated curves used identical parameters and only differed in nitric oxide concentration and mass flow. The simulated curves were superimposed on the measured data and are shown in Fig. 3. The curves correctly replicate key features of the measured data. They show that N_2_(NO: 5 ppb) induced radioluminescence is much less intense than this of N_2_(NO: 50 ppb). They also show that only N_2_(NO: 5 ppb) features a jump-like behaviour upon stopping. The gradual diminishing of the radioluminescence after stopping is replicated by the simulation, too. None of the simulated curves fits the corresponding data perfectly well, however. The imperfect fit is likely due to our very simplified model of the water dynamics. The key concept of the model, however, is well captured. The simulation clearly shows that differences in radioluminescence intensity and stop-flow behaviour can be explained with the dynamics of excitation transfer.

### Remote detection

**Figure 5:**
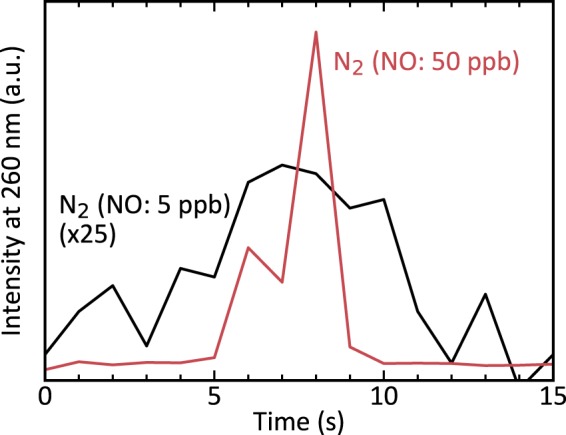
Radioluminescence at 260 nm measured in broad daylight and ambient air. The alpha source has been exposed to a brief but intense nitrogen purge of either N_2_(NO: 5 ppb) (black, 25 times magnified) or N_2_(NO: 50 ppb) (red). For both types of gas, the purge temporarily increased the detected radioluminescence. N_2_(NO: 50 ppb) was about 50 times more effective in doing so.

In Fig. 5 we show that a nitrogen purge can make an alpha emitter produce large amounts of UVC radioluminescence. The source was placed in ambient air, which allowed oxygen and water to quench both a significant fraction of the nitrogen^38^ and almost all of nitric oxide radioluminescence^22,23^. However, by applying a few seconds long lasting nitrogen purge, we created a brief period of increased radioluminescence production. The likely explanation for this behaviour is a reduced influence of quenchers. The purge temporarily replaced the atmosphere around the alpha emitter with NO impurity containing nitrogen, thereby creating an atmosphere similar to this in the tube. Under these conditions, quenchers are absent, and radioluminescence is created more efficiently. When the purge ends, atmospheric conditions turn unfavourable again, and radioluminescence production declines.

The additional radioluminescence produced during the purge consists mostly of contributions from nitric oxide. The stack of interference filters used in this experiment transmitted light around 260 nm^33^ and thus allowed both nitrogen and nitric oxide radioluminescence to reach the PMT. However, nitric oxide is likely to make up the bulk of it, which can be inferred from the observation that a purge with N_2_(NO: 50 ppb) lead to a more than a hundred-fold increase in intensity. In a pure nitrogen atmosphere nitrogen radioluminescence can only be about six times more intense than in air^7,8,15^. Thus, the increase in intensity is likely caused by a vastly increased nitric oxide radioluminescence production.

Purging with nitrogen that contains slightly elevated levels of NO concentration is beneficial for enhancing UVC radioluminescence. The direct comparison of the radioluminescence increase in Fig. 5 shows that N_2_(NO: 50 ppb) is disproportionally more effective than N_2_(NO: 5 ppb) in amplifying the light production, when compared to the respective gases’ nitric oxide content. This disproportionally weak radioluminescence produced by a N_2_(NO: 5 ppb) purge can be explained the same way the low light production of the N_2_(NO: 5 ppb) flow in the tube has been. In Eq. (2) we concluded that nitric oxide radioluminescence approaches maximum light production in an exponential manner with a speed *γ* = *k*_et_[NO]. The higher the NO concentration, the faster the radioluminescence production maximises. In the flow experiment, the radioluminescence from N_2_(NO: 5 ppb) did not fully develop because its NO content was too small for the production to maximise during the brief amount of time the gas was exposed to the source. During a purge, the time the gas remains over the source is even shorter and hence it is to be expected that radioluminescence will not maximise. N_2_(NO: 50 ppb) with a ten times higher concentration of NO did not have those problems and thus showed a disproportionally increased radioluminescence production. This reasoning makes a slightly elevated amount of NO not only beneficial for the additional UVC production, but it also puts less strict requirements on the time the gas needs to be in the active area for purge-induced NO radioluminescence production to be effective.

## Conclusions

In this paper, we demonstrated the production of NO radioluminescence in ambient air. We did so by applying a N_2_ purge that contained NO impurities, which were intrinsic to the gas. It was argued that for such a purge to create NO radioluminescence, the N_2_ gas needs to contain an amount of NO that is dependent on the time the gas remains in the active area. The shorter this time, the more NO is needed. Using N_2_ with elevated levels of NO is generally a good option compared to N_2_ with lower levels of NO. This rule holds as long as self-quenching does not significantly affect the radioluminescence production, which is the case for NO concentrations up to about 50 ppm^28^.

We have shown that NO radioluminescence can be detected in standard indoor lighting conditions using an N_2_ purge that contains NO impurities in concentrations as small as 5 ppb and 50 ppb. The intensity of the produced radioluminescence differs whether the gas is flown over the alpha source or whether it stays static. Under flow conditions, the NO radioluminescence intensity is reduced by the flow due to a limited rate of the energy transfer process. The model explains this behaviour very well, and it can predict the levels of NO that are required in an otherwise pure N_2_ flow to produce intense NO radioluminescence. The model can be used to tailor the application-specific gas atmosphere around an alpha emitter under flow conditions. Concentrations higher than 1 ppm can be harmful for life^40^, so in practice, suitable NO concentrations would be in the range of 100 ppb–1 ppm for safety reasons. The technique can be directly utilised in nitrogen-flushed gloveboxes at nuclear facilities for efficient optical alpha radiation detection in standard lighting conditions.

## Data Availability

The datasets generated during and/or analysed during the current study are available from the corresponding author on reasonable request.
